# A mobile phone intervention to reduce heavy drinking: a preliminary analysis of anchoring heuristics

**DOI:** 10.1186/s40359-023-01465-z

**Published:** 2023-12-06

**Authors:** Jorge Palacios-Delgado, Fabiola Aimeé Guerrero Garduño

**Affiliations:** 1 Universidad del Valle de México, Querétaro, México; 2 Unidad de Investigación en Neurociencias Aplicadas, Querétaro, México

**Keywords:** Anchoring heuristics, Text message, Intervention, Effectiveness, Heavy drinking

## Abstract

**Background:**

Preliminary evidence has been presented for interventions focused on preventing alcohol consumption or reducing the occurrence of episodes of excessive drinking. The anchoring text message intervention is a newly proposed theory-based approach to reducing heavy drinking among youth. The current study tests the preliminary efficacy of this intervention for reducing heavy drinking among Mexican youth.

**Methods:**

Focusing on a sample of sixteen Mexican youths—nine not-heavy drinkers and seven heavy drinkers—the participants completed a survey on alcohol consumption and anchoring heuristics. Pretest and post-test questionnaire data were collected to evaluate the effectiveness of the intervention. The anchoring text messages consisted of strategies to limit alcohol use and feedback reminder messages. Assessments were conducted at baseline, four weeks of intervention, and post-intervention.

**Results:**

Logistic regression analyses indicated a significant effect on heavy drinking episodes post-intervention. The post-intervention anchoring effects among the heavy drinker participants were different from those among the non-heavy drinkers in terms of reporting heavy drinking and future drinking. The anchoring heuristic-based intervention reduced the quantity of drinking and the future estimation of drinking and improved the ability to reject alcohol in the heavy drinker group.

**Conclusions:**

These findings provide preliminary support for the effectiveness of the anchoring heuristic-based intervention, conducted through text messages sent by mobile phone to reduce alcohol consumption. The intervention promoted a reduction in alcohol consumption. Future research should be directed toward investigating the anchoring effects among heavy drinkers.

## Background

A statistic about alcohol consumption among youth an increase was observed [1, 2]. There are various interventions focused on preventing alcohol consumption or reducing the occurrence of episodes of binge drinking. The available evidence on the common interventions (such as cognitive behavioral therapy [3], motivational interviewing [4, 5], internet-based and mobile interventions [6, 7], brief interventions [8–10], and the behavioral economic approach [11, 12] has shown them to be effective in reducing alcohol consumption.

Text messaging interventions have advantages over other forms of intervention, as they have been shown to be effective in modifying the frequency of hazardous drinking in other studies [13, 14]. Further, text messaging offers a more convenient and cost-effective way to support preventive healthcare because it can be scalable at a low cost, can reach large groups of people, and incorporates qualities that are associated with more effective communication interventions, such as tailored messaging, interactivity, personalization, behavioral feedback, and/or high message repetition [15–17].

Recent research using text messaging related to alcohol consumption indicates positive but mixed findings of its efficacy among heavy drinkers [18, 19]. Results in one study showed that text message interventions were effective in reducing alcohol use among college students [20]. Some studies revealed that self-efficacy encourages specific action plans to reduce alcohol consumption [21–23]. The messages can help modify self-efficacy, encouraging individuals to refuse drinks and avoid situations of excessive consumption, maintaining long-term behavior change [24, 25].

The most effective text messages are designed to support behavior change and use feedback as content [26–28]. Recently, a set of interventions was designed to change behavior by altering, modifying, or changing the choice architecture, which involves re-designing the context in which people make choices, to influence decision-making and make certain choices easier [29–31]. A strategy for reducing cognitive biases was presented in the form of a reminder [32]. In some circumstances, when people make rapid decisions (heuristics) in day-to-day functioning, errors can occur, and reminders are often able to reduce these errors. Regarding alcohol consumption, it is crucial that text message interventions are implemented to remind youth of their alcohol limits and their intention to resist the pressure to drink. Experimental research suggests that reminders have been found to be effective even when people have already consumed alcohol [33–35].

Heavy drinking (HD) episodes can result in a high blood alcohol concentration, injuries, car accidents, and impaired decision-making [36]. In previous studies, it has been observed that alcohol reduction interventions are implemented in primary healthcare centers by health professionals and mostly target hazardous and harmful drinkers [37], with sessions ranging from a single 5 to 25 min session to multiple sessions [38] that can be face-to-face or web-based [39]. There are also interventions focused on preventing alcohol consumption or reducing the occurrence of episodes of binge drinking. Research has established the efficacy of interventions that use text messaging, which potentially have the benefit of behavior change. Thus, in our study, we conducted a short-term intervention for alcohol consumption reduction through text messaging, allowing us to observe conclusive changes.

In the context of text message design, behavioral economics based on the anchoring effect can be used to create messages that are more effective at changing behavior. Behavioral economics is a field of study that combines insights from psychology and economics to understand both how people make decisions and how they can be influenced to make better ones [40–42]. It suggests that messages should be framed in a way that emphasizes the benefits of the desired behavior, rather than the costs of not achieving it [43]. Little research has explored the effects of gain- and loss-framed messages on alcohol consumption [44, 45].

Despite the existence of interventions based on behavioral economics [12, 46, 47], there is a lack of knowledge on the effect of anchoring heuristic-based interventions on heavy drinking. Previous research on the anchoring effect is extensive [48–51]. In these studies, it has been shown that anchoring occurs when the anchor values are shown for the estimate of different domains, and it has been suggested that anchors influence behavior and people make decisions under the influence of anchoring [52, 53]. Early explanations of the anchoring heuristic [50, 52, 53] suggest that anchoring values serve as a reference point for people to adjust their response to a range of possible values. Palacios and Guerrero [48] demonstrated that the anchored price of a bottle of alcohol increased the value perceived by consumers and their willingness to pay—they accepted the anchored prices; in this case, the intention to purchase and consume alcohol is more likely.

Inspired by the anchor heuristics and choice perspective, we propose that text messages about alcohol consumption containing an anchoring heuristic can induce individuals to modify their alcohol consumption. For this reason, we incorporated this components within the heuristic text message interventions [49, 53, 54].

The intervention in the present study was built upon behavior change theory based on the behavioral economics design. For the content of the messages, there are decisions in the design of the message that can affect its effectiveness, from the individual message to the pattern of messaging (frequency, duration, and type) and the theoretical approach (anchoring heuristics and the feedback reminder). The research background shows that the intervention proposed in this study may be effective for the prevention of heavy alcohol drinking in different contexts and countries. To our knowledge, a heuristics text-based intervention specifically designed to reduce heavy drinking has not been previously developed.

In the pilot phase, the objective was to evaluate the preliminary efficacy of heuristic-based intervention text messages to reduce heavy drinking among Mexican youth.

We also expected that the participants in the heuristic intervention would show a reduction in alcohol use and heavy drinking and increased self-efficacy for alcohol refusal at the end of the intervention, compared to the baseline condition.

## Methods

### Design

We conducted a pilot study to inform the development of an intervention based on behavior economics to reduce heavy drinking among young people via WhatsApp text messaging. In the pilot phase, we examined the feasibility of the proposed intervention. We gathered initial evidence of the efficacy by examining the changes in heavy drinking between the baseline, intervention, and follow-up.

### Participants

Participants were recruited in August 2022 and were required to be 18 years or older and have daily access to the Internet. Recruitment was conducted online, through advertisements on social media, especially WhatsApp. The sample consisted of 16 young people (50% women and 50% men, with an age range between 19 and 25 years (*M* = 22.12; *SD* = 2.0)), selected via a non-probabilistic sampling approach. The sample was predominantly from Querétaro State in Mexico. The participants reported having at least a bachelor’s degree. Of the participants, 43.7% studied, 31.3% worked, and 25% studied and worked. The eligibility criteria for participants were that they were at least 18 years old, had a mobile phone, used social networks, and were interested in evaluating text messages regarding alcohol consumption. If the participant met the eligibility criteria, the researcher contacted them, explained the project procedures and confidentiality, and invited the participant to join a WhatsApp group.

### Measures

Data on alcohol use were collected both at baseline and at follow-up. For the heuristic messages, each week the participants were asked about the estimated quantity of alcohol they consumed (susceptibility to anchoring), and they responded to the interest and persuasiveness of each text.

#### Alcohol consumption

Drinking was measured by four questions on the age of onset of alcohol consumption, the frequency of alcohol use in the last month (from never to daily), the quantity of alcohol consumed per event (number of drinks consumed per occasion), the incidence of heavy alcohol drinking (five or more glasses per event; from never to daily), and the number of drinks they think they need to become drunk (number of glasses of alcohol). Participants reported the mean number of standard drinks consumed on a single occasion in the past weeks. Higher scores indicate a higher quantity of drinking [55].

Heavy drinking was considered based on the number of standard glasses they reported drinking; for men, it was five standard glasses, and for women, it was four, per consumption occasion. This measurement has been proven in several studies using Mexican samples [55, 56], and the Mexican sociocultural context [57] was used in the present study.

#### Anchoring heuristic

We adapted the task used by Jacowitz and Kahneman [58] to measure the quantity of alcohol consumed in anchoring. In the original study, the authors asked about the length of the Mississippi River (in miles), and the experimental subjects answered first whether the quantity was greater or less than an anchor value, and then they estimated the quantity and indicated their confidence in the estimate. For our research, with the use of an open-question format, participants were first asked about the quantity of alcohol they proposed to consume. The questionnaire used here has been previously tested in studies in Mexico [48].

Congruent with the typical anchoring procedure, in the first question, participants were presented with a hypothetical anchoring task involving the quantity of alcohol they proposed to consume. This initial activity served as the “anchor”. Immediately, all participants were then asked, “The next time you drink alcohol, how many standard glasses of alcohol would you like to drink?”.

#### Alcohol self-efficacy

Self-efficacy was assessed with a single self-report item. Participants rated on a scale of 1 (no confidence) to 10 (very confident) how capable they were of refusing to consume alcohol at a meeting, party, or club. This single item was obtained from the self-efficacy scale for risky behaviors [23], which has been used with good results in Mexican samples [59, 60].

### Procedure

The intervention was based on sending a series of text messages to which the participants were exposed over a four-week period and a follow-up, during the weekends. These messages were written in the language of the culture (e.g., expressions such as “Hey!” were used to say hello, or “We hope you had an incredible time to end the week”) of the target age group. The content of the messages was constructed over three main phases during the week (Thursday, Saturday, and Sunday): each Thursday, participants were sent a text message with a heuristic, each Saturday they were sent a reminder, and each Sunday they were asked about their consumption after drinking over the weekend.

At baseline, the participants were required to answer a form asking about their regular alcohol consumption. An introductory text welcoming participants and explaining the purpose of the study was sent. Following the baseline survey, participants received a total of eight messages over the next four weeks. The content of the intervention included two strategies (two types of messages): the first consisted of texts that attempted to modify alcohol consumption through a heuristic, for example, “A group of young university students your age proposes moderating alcohol consumption in bars and clubs to four standard drinks or glasses for men and women.” The second strategy consisted of reminders. These reminders included strategies to limit drinking as well as positive and normative feedback; for example, “Here are a few ideas to take care of yourself if you are going out to a party, bar, or meeting today. Keep track of how many glasses of alcohol you drink and separate them with soda or water, eat before attending your event, and if necessary, have a sober driver ready or contact transportation services by app.” Finally, all subjects were asked to complete a follow-up form one month after the baseline assessments.

## Data analysis

Baseline descriptive characteristics of the overall sample were obtained, including demographic information (sex, age, occupation) as well as the means and standard deviations for the alcohol consumption (frequency of alcohol use, number of drinks consumed per occasion, heavy alcohol drinking, and number of drinks for drunkenness). Mann–Whitney U tests were used to determine the differences in drinking behavior between the HD and not-HD groups. To assess differences in drinking behavior between the pre- and post-intervention assessments, we used the Wilcoxon matched-pairs signed-rank test.

In addition, to assess the effectiveness of the intervention, logistic regression modeling was performed to determine whether the post-intervention assessment and follow-up were significantly different from the baseline measurement on the alcohol-related variables.

Throughout all the analyses, *p* ≤ 0.05 was interpreted as statistically significant. Effect sizes (for the mean differences between the baseline, post-intervention, and follow-up) were reported as *Hedges g,* *Glass Δ,* and *r* (r = z/√n) for nonparametric data and interpreted using the conventional metrics as small = 0.10, medium = 0.30, and large = 0.50 [61].

## Results

Based on their consumption levels, the participants were divided into two groups: (1) not heavy drinkers (low consumption drinkers, fewer than four standard glasses of alcohol for women and five or fewer for men) and (2) heavy drinkers (for women, this was classed as 4 or more standard glasses of alcohol on one occasion in the previous 30 days; for men, the number of drinks was 5 or more). In total, 16 participants were assigned to either the non-HD group (9 participants: 4 women and 5 men) or the HD group (7 participants: 4 women and 3 men).

Of all the participants, 87.58% drank alcohol, starting at age 15.44 (*SD* = 1.9) years, and 43.8% had a frequency of once a month or fewer. The heavy alcohol consumption (> 5 glasses in a single instance) rate was 50.0%. The range of drinks consumed varied between 1 and 15 (*M* = 5.13; *Md* = 5.0; *SD* = 3.3). In addition, the participants reported the number of glasses they considered necessary to become drunk, reporting a mean consumption of 6.5 (*Md* = 7.50; *SD* = 2.6) drinks.

At baseline, there were differences between the HD and the non-HD groups in quantity (*U* = 1.000; *Z* = –3.26; *p* < 0.01; *Glass* *Δ* = 1.27), previous drinking behavior (*U* = 0.500; *Z* = –3.32; *p* < 0.001; *Glass* *Δ* = 1.23), and future drinking (*U* = 9.000; *Z* = –2.24; *p* < 0.05; *Glass* *Δ* = 1.13). To analyze the differences between the HD and non-HD groups post-intervention, we examined the results of the quantity, episodes of heavy drinking, previous quantity consumed, and estimated quantity of alcohol in the future. These results are summarized in Table 1. Significant differences were revealed between the participants in the non-HD group versus the HD group in quantity (*U* = 8.000; *Z* = –2.59; *p* < 0.05; *Glass* *Δ* = 1.58) and self-efficacy (*U* = 13.000; *Z* = –2.10; *p* < 0.05; *Glass* *Δ* = 1.35).

**Table 1:** Alcohol drinking behavior responses in the heuristic intervention by heavy-drinking status

	Pre-intervention				Post-intervention			
	Non-HD		HD		Non-HD		HD	
	*M*	*SD*	*M*	*SD*	*M*	*SD*	*M*	*SD*
Quantity	3.22	1.6	7.57	3.4	2.77	1.6	5.00	1.0
Binge drinking	5.78	3.0	7.43	1.9	6.22	3.2	8.28	2.8
Future	2.75	1.4	5.14	2.1	2.33	1.7	3.13	1.2
Previous drinking	2.22	1.5	7.86	4.5	3.1	2.6	4.72	2.6
Self-efficacy	8.88	1.8	5.57	3.5	9.5	0.7	6.70	3.0

*non-HD* not heavy drinkers, *HD* heavy drinkers, *M* mean, *SD* standard deviation

The primary goal of this intervention was to minimize heavy drinking among youth, and we examined the influence of the intervention on those participants who were heavy drinkers at baseline. After the intervention with text messages based on heuristics, the analyses conducted among the participants identified as heavy drinkers showed an effect of the intervention on the quantity (*W* = 15.00; *Z* = –2.03; *p* < 0.05), future drinking (*W* = 15.00; *Z* = –2.04; *p* < 0.05), and self-efficacy (*W* = 6.00; *Z* = –2.06; *p* < 0.05). The effect size varied across drinking indicators (quantity (*Hedges* *g* = 3.69; *r*_*B*_ = 0.87), future drinking (*Hedges g* = 1.63; *r*_*B*_ = 0.72), and self-efficacy (*Hedges g* = 1.14; *r*_*B*_ = 0.73). Among non-drinking participants, post-intervention, the results did not show any impact of the intervention on alcohol drinking.

Finally, we examined the influence of the intervention on alcohol drinking indicators as a dichotomous variable based on non-HD and HD. Table 2 provides details of the regression coefficients used in the logistic regression model. At baseline, the logistic regression model was statistically significant. The logistic regression model was statistically significant (χ^2^ (14) = 19.157, *p* < 0.001). The previous drinking was associated with an increase in alcohol consumption for HD, with an *R*^2^ Nagelkerke value of 0.93.

**Table 2:** Influence of the intervention on alcohol indicators between the non-HD and HD groups

Model ^a^	*b*	*SE*	*β*	*Odds Ratio*	*CI* = *95%*
*Baseline*					
Previous drinking	21.20	10,918.08	89.87	1.62	0.29–2.0
*Post-intervention*					
Quantity	45.47	19,984.27	80.48	5.59	1.1–9.7
Future drinking	–55.65	25,601.13	–85.53	6.77	0.99–7.7
Self-efficacy	–15.13	7920.04	–37.77	2.67	0.94–3.4

^a^Coded as 0 = non-HD, 1 = HD. *p < 0.05, **p < 0.01, ***p < 0.001. *CI* confidence interval, *SE* standard error

Post-intervention, we also assessed the logistic regression model as statistically significant (χ^2^ (12) = 5.253, *p* < 0.05). The model correctly classified 93.75% of the cases. Analysis of our data showed that the quantity of alcohol consumed was associated with more than three but fewer than five drinks per drinking occasion in the HD group, while a decrease in the future estimate of their consumption and a reduction in their self-efficacy affected the quantity of alcohol consumption in the HD group. The incorporation of the intervention based on the anchoring heuristic reduced the future estimation of alcohol drinking by 6.7 times and the probability of having a lower self-efficacy to reject alcohol by 2.6 times in the HD group. The Nagelkerke *R*^2^ value was 0.93, indicating that approximately 93% of the variability in the consumption in the HD group was explained by the quantity, the future estimation of consumption, and the self-efficacy.

## Discussion

This study tested the effectiveness of an intervention using heuristic-based alcohol consumption text messages for reducing heavy drinking among Mexican youth. It is one of the first studies to integrate text messages and heuristics. Thus, this is an emerging field for carrying out interventions on similar topics.

Regarding the evaluation of the baseline, differences were found between the HD and non-HD groups, and post-intervention in the quantity of alcohol consumed and self-efficacy among the groups. Comparing the reduction in the quantity of alcohol consumed, the non-HD group showed similarities in their alcohol consumption; however, despite that the HD group had a higher consumption than the non-HD group post-intervention, the latter reported a decrease in the number of glasses of alcohol consumed. The challenges were more pronounced in quantity, previous drinking, and future consumption among the HD participants, and we also observed reductions in these measures among the non-HD group. This finding is similar to other studies [18, 26, 28], which have documented text message content that was applied to specific drinking contexts, including messages for before and after a drinking occasion and messages that are tailored to different drinking habits.

The existence of differences in self-efficacy between the HD and non-HD groups post-intervention shows an indirect effect through the modification of the ability to reject alcohol consumption. Thus, this finding is promising for modifying self-efficacy through heuristics. Changes in socio-cognitive factors (self-efficacy) mediating changes in alcohol use also improved in the intervention group, consistent with recent reports about self-efficacy [18, 21, 24, 25], indicating that the purpose of the intervention was achieved.

For this study, our data confirmed the hypothesis, which proposed that participants in the heuristic intervention would show a reduction in alcohol use and heavy drinking and increased self-efficacy for alcohol refusal, compared to the baseline condition. Our results provide preliminary evidence that the text message intervention, based on heuristics, was helpful in reducing the quantity of alcohol consumed by the participants in the study and maintaining an increase in self-efficacy. This is consistent with previous studies testing alcohol interventions using text messages [6, 7, 25, 26].

This is the first study in Latin America to empirically test a text messaging intervention theoretically based on the anchoring heuristic for reducing heavy drinking among youth. This finding is particularly important in that the heuristic intervention provided text messages that gave specific information about using these strategies to limit drinking.

Here, the discussion focuses on how the content of the message works in situations of alcohol consumption using numbers that serve as an anchor. In previous studies [60], we reported how young people estimate the quantity of alcohol to consume after a text with a number (anchor) has been received. In this study, we demonstrated that the anchored value is relevant to reduce their present and future consumption. Based on these findings, we incorporated the anchor number into the content of the message, which was fewer than five—the equivalent of the level of excessive alcohol consumption. Therefore, for the anchoring heuristic to have an effect on the participants, the consumption levels after the anchoring (post-intervention) would have to be fewer than five drinks or standard glasses. Our data confirmed that the quantity of alcohol drinking in the HD group (pre-intervention: *M* = 7.57 versus post-intervention: *M* = 5.00) and the estimate of future drinking (pre-intervention: *M* = 5.14 versus post-intervention: *M* = 3.13) decreased after the introduction of text messages, suggesting that the participants adopted some of these strategies (anchor heuristics and feedback) to limit their alcohol consumption. This is similar to the findings of previous studies [18, 20, 26–28] on text message interventions, which revealed reductions in alcohol. Our findings complement the existing literature describing research on how to design text message interventions for binge drinking prevention.

This was the first text messaging study to incorporate mobile messaging for heavy drinking behavior. Previous evidence has shown that anchoring effects are very reliable in changing a decision [48, 49, 53, 54, 60], and by incorporating numerical anchors, we modified the decision to consume less alcohol, reducing consumption among young people.

The results of the logistic regression analyses of the participants provide evidence of the value of this approach (anchoring heuristics) as an intervention strategy to minimize heavy drinking among young people. Consistent with previous studies [6, 7, 25, 26] using text messages to reduce alcohol drinking, we found that post-intervention, text messages based on the anchoring heuristic were associated with a lower likelihood of heavy drinking, a decrease in future consumption, and an increase in self-efficacy among HD young people. Our findings complement the existing literature describing research on how to design text message interventions for heavy alcohol use prevention.

We decided to use the text message intervention in this research because the existing literature indicates that text messages sent via mobile phones are effective [13, 14, 18, 19, 24, 27, 28]. We found similar results in our study in the sense that there was a decrease in alcohol consumption. This could be because this type of alcohol intervention can provide better results, reach a larger number of people, and be performed in a more cost-effective and accessible way, as well as reach young adults in the environment in which they are making decisions about their alcohol consumption [15–17, 25, 26]. This ease of use and the flexibility of text messaging to send and receive messages anytime and anywhere, as well as the ability to receive tailored information, could be responsible for the effect of this intervention.

Although these results are promising, the present study has several limitations. First, our sample was not representative of the entire country since it was a pilot study; thus, further research should be conducted with more representative samples. Additionally, a sample size of 16 is very small, especially since there were only 7 heavy drinkers. Although the sample size was small, this pilot study provides valuable preliminary data that can inform broader future research. The pilot study also had a smaller sample size because the feasibility of the anchoring heuristic in text messages was evaluated. We recognize that the conclusions that can be drawn from this study are limited due to the small sample size, and additional studies with larger sample sizes are necessary to confirm our findings.

Second, in the post-intervention condition, among the HD group, there was an increase in excessive drinking. We considered that this was because, in the last week of the intervention, there was a holiday (Mexican Independence Day), during which it is common to consume large amounts of alcohol; thus, the quantity of drinking during that weekend increased. Third, the content of the messages was constructed using heuristic-based text messages and reminders, and we are uncertain which of the two may have been more significant, the heuristic alone or combined with a reminder. We believe that in future studies, it is necessary to carry out an intervention using only text messages based on the anchoring heuristic, without reminders, to distinguish the independent effects of anchoring and reminders as individual strategies for behavior change in alcohol consumption.

Fourth, considering that the information collected was self-reported, it is possible that there was a social desirability bias, and some participants may have exaggerated or underreported their alcohol consumption. Fifth, correctly remembering how much alcohol they drank was another limitation, as some participants may have had difficulty accurately recalling the number of drinks consumed, especially if they were drunk. Finally, a replication of this result is needed in other samples to confirm the effect of text messages on alcohol consumption in young people.

### Implications for future research

Among the strengths of this research, it is the first study with these characteristics carried out in Mexico and Latin America to empirically test a text messaging intervention theoretically based on the anchoring heuristic for reducing heavy drinking among youth. We observed that it was possible to carry out these interventions for a shorter period and develop them through different applications containing messaging services and still obtain favorable results, in contrast to other studies. Finally, for the continuity of the present study, we proposed interventions based on economic behavior, by incorporating the framing effect, to elaborate on the content of text messages that can be implemented in a future intervention to reduce alcohol consumption among young people.

## Conclusions

Our results support the efficacy of using anchoring heuristic-based text messaging in reducing heavy drinking and increasing self-efficacy among youth. Such approaches may provide an alternative mode of reducing the occurrence of episodes of binge drinking that can be integrated with other secondary prevention efforts. The post-intervention findings among HD participants showed the effects of the intervention on alcohol consumption. In the HD group, participants decreased the estimation of their future alcohol consumption, and the impact of self-efficacy on the quantity of alcohol consumption was increased. This study supports the idea that behavior change can be achieved as a result of engaging in mobile text interventions based on behavior economics.

## Data Availability

All data and text material are available upon request from the first author.
